# A randomized controlled trial of a group-based gaze training intervention for children with Developmental Coordination Disorder

**DOI:** 10.1371/journal.pone.0171782

**Published:** 2017-02-10

**Authors:** Greg Wood, Charlotte A. L. Miles, Ginny Coyles, Omid Alizadehkhaiyat, Samuel J. Vine, Joan N. Vickers, Mark R. Wilson

**Affiliations:** 1 Centre for Health, Exercise and Active Living, Manchester Metropolitan University, Crewe, United Kingdom; 2 Department of Health Sciences, Liverpool Hope University, Liverpool, United Kingdom; 3 College of Life & Environmental Sciences, University of Exeter, Exeter, United Kingdom; 4 Faculty of Kinesiology, University of Calgary, Calgary, Canada; TNO, NETHERLANDS

## Abstract

The aim of this study was to integrate a gaze training intervention (i.e., quiet eye training; QET) that has been shown to improve the throwing and catching skill of children with Developmental Coordination Disorder (DCD), within an approach (i.e., group therapy) that might alleviate the negative psychosocial impact of these motor skill deficits. Twenty-one children with DCD were split into either QET (8 male 3 female, mean age of 8.6 years (*SD* = 1.04) or technical training (TT) groups (7 male 3 female, mean age of 8.6 years (*SD* = 1.84). The TT group were given movement-related instructions via video, relating to the throw and catch phases, while the QET group were also taught to fixate a target location on the wall prior to the throw (QE1) and to track the ball prior to the catch (QE2). Each group partook in a 4-week, group therapy intervention and measurements of QE duration and catching performance were taken before and after training, and at a 6-week delayed retention test. Parental feedback on psychosocial and motor skill outcomes was provided at delayed retention. Children improved their gaze control and catching coordination following QET, compared to TT. Mediation analysis showed that a longer QE aiming duration (QE1) predicted an earlier onset of tracking the ball prior to catching (QE2) which predicted catching success. Parents reported enhanced perceptions of their child’s catching ability and general coordination in the QET group compared to the TT group. All parents reported improvements in their child’s confidence, social skills and predilection for physical activity following the trial. The findings offer initial support for an intervention that practitioners could apply to address deficits in the motor and psychosocial skills of children with DCD.

***Trial registration*: ClinicalTrials.gov** NCT02904980

## Introduction

Developmental coordination disorder (DCD) is a condition estimated to affect around 6% of children [1]. The condition is categorised as a marked impairment in the development of motor coordination that interferes with activities of daily living below the level expected for the child’s chronological age, which must not be attributable to neurological conditions, sensory problems or low intelligence [2]. While the aetiology of DCD is still poorly understood, children with DCD suffer motor deficits related to internal (forward) modelling, rhythmic coordination, executive function, gait and postural control, catching and interceptive action, and sensoriperceptual function [3].

In an effort to understand more about the mechanisms behind such deficits, researchers have explored the role and control of vision in children with DCD, compared to typically developing (TD) children. A body of evidence has linked DCD to significant impairments in general visuomotor control and the processing of task-relevant, visual information [3]; the ability to use predictive information to guide action [4]; the pursuit tracking of objects [5]; and the ability to maintain fixation on visual targets [6]. While some of this research is laboratory-based, these deficits in the control of vision have obvious implications for the production and control of coordinated movement in the ‘real-world’. For example, the ability to maintain a fixation on a visual target and track an object is fundamental for aiming and interception skills that are the building blocks for activities in sport and playground games.

Throwing and catching is a perfect example of a task where these visual abilities are critical and it is unsurprising that children with DCD find this task difficult. In a recent study [7], we explored the visual control of children in a task that required them to throw a ball against a wall and subsequently catch it. In this study we measured–using a measure of optimal visual control derived from sport (the quiet eye (QE) [8])—the duration of time children spent fixating a target before throwing (QE1) and the duration of time spent pursuit tracking the ball prior to the catch attempt (QE2). These QE durations provide periods of pre-programming that assist with thrower’s predictions of the location of the bounce point on the wall and subsequent location and timing of the interception point of the catch [7,9]. Results suggested that highly proficient children demonstrated longer QE aiming fixations (QE1) before the release of the ball and longer QE pursuit tracking durations (QE2) on the ball prior to the catch. Mediation analyses revealed that the superior performance of the high motor proficient children was underpinned by an earlier and longer QE2 before the catch attempt [7].

Interestingly, in further studies we have demonstrated that teaching participants to adopt the QE strategy of skilled children through observing video footage of their eye-movements (QE training; QET), improved catching technique in children with average motor skill ability [9,10] and children with DCD [11]. In the latter study, DCD children who were given a brief QET intervention experienced significant improvements in their catching coordination and catching kinematics. Moreover, these benefits were maintained even after a 6-week detraining period. A control group, who received typical movement-focused instructions, revealed no improvement in catching technique at the 6-week delayed retention test [11]. The authors concluded that QET served to improve the attentional control of these children, providing more optimal aiming behaviour (QE1) and more time to track the early flight phase of the ball as it came towards them (QE2). As impairments in the ability to maintain target-focused fixations and pursuit tracking of objects has been shown to be characteristic of DCD [6], we believe that QET, a strategy that has been shown to teach this form of top-down attentional control [8], may be effective in the treatment of this condition.

The motor deficiency associated with DCD also has severe consequences for the psychosocial development and wellbeing of the children who suffer from it. For example, children with DCD report being excluded (by either their peers or themselves) from partaking in physical activities or playground games [12]. This exclusion has an additive negative effect on their psychosocial development, resulting in children with DCD experiencing loneliness, victimisation and feelings of rejection by their peers [13] and lower levels of confidence, self-esteem and self-worth [14].

In an attempt to alleviate the tendency for social exclusion, studies have examined the effectiveness of group-based training interventions for children with DCD. Group-based therapy might not only be more effective than individual-based therapy in improving motor skills in children with DCD [15], but may also improve psychosocial variables [13]. Furthermore, not only is group therapy more time efficient and cost effective for the therapist, parents of children with DCD emphasise the importance of their children being able to participate in organised, physical activity groups and value therapy that improves their child’s perceptions of self-confidence and competence over those that just focus on improving motor abilities [16]. These views have led to suggestions that therapeutic interventions should therefore focus on enhancing the social, as well as the physical, skills of children with DCD [17].

The aims of this study were two-fold. First, we sought to build upon Miles et al.’s positive findings for QET for children with DCD [11] by adopting multiple sessions of training, in an attempt to increase the training effect and to more closely resemble typical therapy. We hypothesised that QET would significantly improve the catching performance of children with DCD compared to a group receiving typical, technical instructions by improving their visuomotor control. Specifically, we hypothesised that QET would facilitate more optimal aiming behaviour (longer aiming durations prior to the throw; QE1) and quicker and longer tracking durations of the ball prior to the catch (QE2). Furthermore, we hypothesised that these visuomotor improvements for the QET group would mediate the predicted performance advantage in catching for QET children. Specifically, mediation analysis should reveal that a longer QE aiming duration (QE1) predicts an earlier onset of tracking the ball prior to catching (QE2) which predicts catching performance [9].

A second aim was to explore the feasibility and effectiveness of using a group-based therapy approach as a framework for the delivery of this intervention. We hypothesised that regardless of the intervention received, parents would report that their children found the intervention to be more beneficial than typical, individual, therapy and would consequently report increases in their children’s confidence, enjoyment and predilection for sport or physical activity.

## Methods

### Participants

Twenty-one children, aged 7–11 years old, were recruited from local DCD support groups, social media, and local occupational therapy centres in the North West of England. Recruitment and follow-up took place between February 2015 and September 2015. All children scored below the 5^th^ percentile on the Movement Assessment Battery for Children-2 [18] (MABC-2) carried out at the baseline testing phase. Using each child’s baseline score from the MABC-2 throwing and catching task, two randomised groups were formulated, by the second author. The QET group (8 males and 3 females) and the TT group (7 males and 3 females) were matched for age and MABC-2 score in a parallel group design (Table 1).

**Table 1:** Demographic and clinical characteristics (means and SD) of the Quiet Eye Training (QET) and Technical Training (TT) groups.

	QET	TT	*p*
Age (yrs.)	8.55 (1.04)	8.60 (1.84)	.993
MABC-2%	2.55 (2.09)	1.93 (2.19)	.552
ADHD %	87.91 (17.10)	90.50 (14.70)	.721

**Fig 1:**
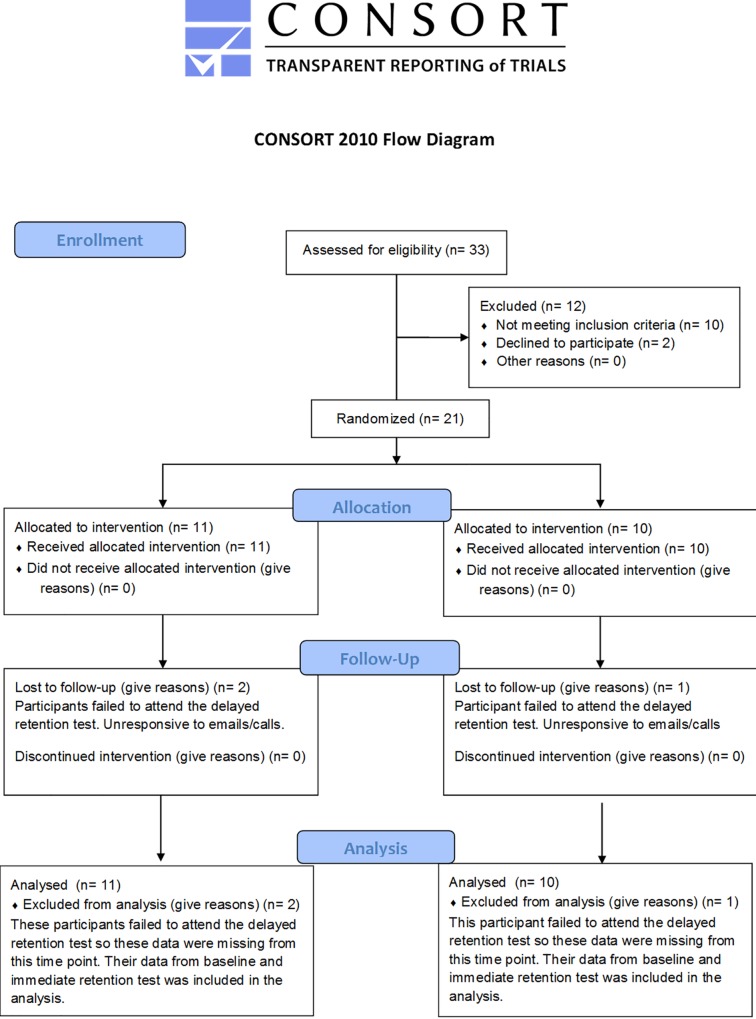
A CONSORT flow diagram outlining participant recruitment and analysis.

Children and their parents were blinded to their intervention allocation. Parents also completed the Attention Deficit/Hyperactivity Disorder (ADHD) Rating Scale-VI [19] prior to testing. No child scored above the 98^th^ percentile for inattention or hyperactivity, which is recommended to be the minimum cut-off used as an indication of ADHD in research [19]. All children were classified as of ‘normal’ intelligence based on their teacher/parent reports. UK NHS ethical approval (15/NW/0279) was granted by the RES Committee North West—Greater Manchester South, before any testing was carried out, and parents and children provided written informed consent before taking part. Fig 1 shows a CONSORT flow diagram outlining participant recruitment and analysis (see also S1 Fig CONSORT checklist and the original study protocol in S1 File). Due to an oversight, the study protocol was not registered as a clinical trial before enrolment of participants but the authors confirm that all ongoing and related trials for this intervention are registered (ClinicalTrials.gov Identifier: NCT02904980).

### Throwing and catching task

The throwing and catching task from the MABC-2 (8–10 year age bracket) was used to aid comparison with previous studies [8, 9, 10, 11]. It requires participants to stand behind a line situated 2 metres from a wall, throw a tennis ball against the wall using an under-arm action, and catch it before it bounces. In line with MABC-2 instructions, the task was first explained to the participant and demonstrated once, before the participant took five practice attempts.

### Apparatus

Each participant was fitted with an Applied Science Laboratories’ Mobile Eye XG gaze registration system (ASL, Bedford, MA), measuring point of gaze at 30 Hz. A 30 Hz Digital SLR camera (Finepix S6500fd) was placed on a tripod 2 metres to the right of the throw line at shoulder height of the participant. This captured a side-on view (sagittal plane) of the participant’s movements during the throw and catch action. Additionally, children were fitted with four upper limb 3D inertial motion capture sensors (two on each arm) and 11 surface EMG electrodes to the upper and lower body muscles (Noraxon, USA). The analyses of detailed kinematic and muscle activity data will be submitted in a separate paper.

### Procedure

Testing was divided into baseline, training, retention and delayed retention phases. During the baseline phase, children attended the laboratory individually, and following the completion of written consent, completed the MABC-2 protocol. Prior to completing the throwing and catching task, each participant was calibrated to the eye-tracker using nine locations on the wall to which he/she were required to throw the ball against. They then completed five blocks of 10 trials of the MABC-2 throw and catch task. Participants were then pseudo-randomly divided into two experimental groups based on this initial throwing and catching performance. The procedure for the retention and delayed retention phases replicated the baseline phase. The retention phase occurred 1 week after the end of the training phase (week 6) and the delayed retention phase took place 6-weeks after this point. During the delayed-retention phase, parents completed the parental feedback questionnaire while their child completed the throwing and catching task procedure. All parents and children were blind to their group allocation.

### Training protocols

The training phase consisted of each group attending the university sports hall on separate days during four weekly (1hr) group sessions (See Table 2). At the beginning of each session, each group watched a brief instructional video showing the same expert model completing the throwing and catching task. Both videos provided a split screen of the model showing a side-on view of their movement and a first person view taken from the eye tracker, showing the point of gaze while performing the task. The videos were edited to reinforce the different training instructions. The TT group videos highlighted the movement of the expert model whereas the QET group videos highlighted the gaze footage of the same expert model [11] (see S2 File). The individual in this video has given written informed consent (as outlined in PLOS consent form) to publish these case details.

**Table 2:** A week-by-week breakdown of the training activities and instructions for Quiet Eye Trained (QET) and Technically Trained (TT) groups.

Week	Activities	QET Instructions	TT Instructions
**Week 1 –Accurate Throwing**	• 20 warm up MABC-2 throw and catches	Focus your eyes on the target and count to two before you start a smooth throwing action	Throw at a target using a smooth throwing action.
	• Watch instructional video for throwing		
	• Target-related activities (throwing bean bags into buckets, throwing balls at cricket stumps, throwing at cut-out faces stuck on a wall).		
	• Started at short distances and increase distance based on individual success		
	• Competitive team games using the same tasks		
	• De-brief and reinforced instructions		
**Week 2 –Effective catching**	• 20 warm up MABC-2 throw and catches.	Keep your eye on the ball until it comes back into your cupped hands	Concentrate on the ball and cup your hands together.
	• Watch instructional video for catching		
	• Catching-related activities (catching large sponge balls and beanbags, catching with a bucket instead of their hands, catching while moving around).		
	• Varied distance and speed of the catch		
	• Competitive team games using the same tasks.		
	• De-brief and reinforced instruction		
**Week 3 –Linking throwing and catching**	• 20 warm up MABC-2 throw and catches	Questioning on previous instructions and instruction on combining coaching points together	Questioning on previous instructions and instruction on combining coaching points together.
	• Watch instructional video linking the throw and catch		
	• Throwing and catching tasks (throwing and catching between participants while walking around, throwing and catching a ball along a chain, rounders with a larger sponge ball where children hit the ball with their hands rather than a bat)		
**Week 4 –Throwing and catching competitive games**	• 20 warm up MABC-2 throw and catches	Questioning on previous instructions and reiteration of coaching points	Questioning on previous instructions and reiteration of coaching points
	• Children chose their favourite games from the sessions and were prompted to remember the related coaching points		

After watching each video participants were questioned regarding its content to check their understanding. Participants then completed 20 ‘warm-up’ trials of the MABC-2 throwing and catching task in unison, while coaches reinforced the respective coaching instructions. Throughout the intervention both groups took part in exactly the same throwing, catching and related interception type activities but the instructions that were emphasised by the coaches were different for each group. In short, the TT group received technique-based instructions related to the mechanics of the skill, taken from a UK physical education resource [20]. The QET group received instructions that were related to controlling their eye movements so that they tracked the ball for longer [9,10,11].

### Measures

#### Quiet Eye

QE measures were analysed using Quiet Eye Solutions software (www.quieteyesolutions.com). QE1 was defined as a targeting fixation located on the target on the wall (that remained within 1° of visual angle for more than 100ms) prior to and during the throw phase of the task. Onset of QE1 was defined as the final fixation duration prior to the initiation of foreswing of the throwing arm and offset occurred when gaze deviated off the target by more than 1° of visual angle for longer than 100ms [11]. QE2 was defined as the final tracking gaze on the tennis ball after it rebounded from the wall during the catch phase of the task. QE2 onset was defined as the start of the final tracking gaze on the ball (for more than 100 ms) before the grasping action was attempted, or the trial ended. QE2 offset occurred when the tracking gaze deviated off the ball for more than 100ms [11].

#### Catching performance

Catch success was scored using the sagittal motor video footage, and expressed as the total number of balls successfully caught out of 50 attempts. A measure of catching quality was also determined from the video footage providing a more sensitive measure of catching performance. Whereas catch success is binary (only a perfect catch can be considered successful), a technique score recognises differences in the quality of the attempt to catch, and awards more points to better attempts. For example, using the binary catching success measure, children who make no reaction to the ball as it comes back to them are awarded the same score (0) as a child who fumbles the ball or manages to catch it by trapping it against their arms. The catching performance scale recognises and quantifies these distinct differences in catch attempts. The catching performance scale (Table 3 [11]) consisted of an 11-point scale whereby catch attempts were given a score between ‘0’ (Makes no move towards the ball as it comes back) and ‘10’ (The catch is made exclusively with the palms and fingers). A second blinded researcher scored 10% of the trials to check for inter-rater reliability (92%).

**Table 3:** The qualitative catching performance scale.

Outcome	Code	Description
No reaction	0	Makes no move towards the ball as it comes back
Reaction, no contact	1	Makes some move towards ball, no contact, no attempt at a catch (delayed)
Inaccurate/delayed reaction, no contact	2	Reacts to ball direction and makes effort to catch the ball.
Delayed reaction, no contact before bounce	3	Reactions to ball direction and makes effort to catch the ball. Ball bounces/contacts some part of the body
Delayed reaction, limited contact	4	Reacts to the ball, poor throw results in it bouncing/contacting another surface before catch can be made
Ball contacts hands	5	The ball contacts one or both hands but there is no control
Trap ball, no hands	6	Ball hits body and trapped with arms but not hands
Fumble	7	Ball is fumbled and drops to the ground
Trap	8	The ball is grasped by both hands, with the aid of the trunk or other body part
Fumble but re-grasped	9	Clean catch completed after a fumble without ball hitting another surface
Clean controlled catch	10	The catch is made exclusively with the palms and fingers

#### Parental questionnaire

The questionnaire consisted of two parts. Part I consisted of five 5-point Likert scale questions (1 = not at all; 3 = somewhat; 5 = very much so) relating to motor skill improvements, confidence and changes in their child’s predilection for physical activity. Part II consisted of three short answer open-ended questions that asked parents to document any changes they may have noticed post training; to list any aspects of the training that their child particularly enjoyed; and a final question that asked for any further comments about any aspect of the intervention.

### Data analysis

All statistical tests were analysed using IBM SPSS version 22. Based on per-protocol analysis approach, QE and performance variables were subjected to mixed design 2 (Group: QET vs TT) x 3 (Phase: baseline, retention and delayed retention) ANOVA. Effect sizes were reported using partial eta squared statistics and sphericity assumptions were not violated for any variable. Based on our hypotheses, we focus our discussion on significant interactions (when found), which were followed up using repeated measures ANOVAs and Bonferroni corrected pairwise comparisons where appropriate. Mediation analyses were computed using the MEDIATE SPSS custom dialog [21]. For this, and in line with theorising by Miles et al [9], delayed retention catching success was entered as the dependant variable and QE1 duration was entered as the independent variable. QE2 onset was entered as the proposed mediator. This process allows inferences to be made about the indirect effects using percentile bootstrap confidence intervals.

Parental questionnaire data (Part I) were analysed using Mann-Whitney *U* tests and 95% confidence intervals. Content analysis was used to analyse the free response statements (Part II) from the open-ended questions [22]. This involved two researchers reading each parental response and categorising key themes that occurred. Themes were then combined into five high-order themes through a process of discussion, before quantifying the frequency of these responses.

## Results

Three participants (2 QET and 1 TT) dropped out of the study and failed to complete the delayed retention test. Consequently, three parents (2 QET and 1 TT) also failed to complete the parental questionnaire at delayed retention.

### Quiet Eye

**Fig 2:**
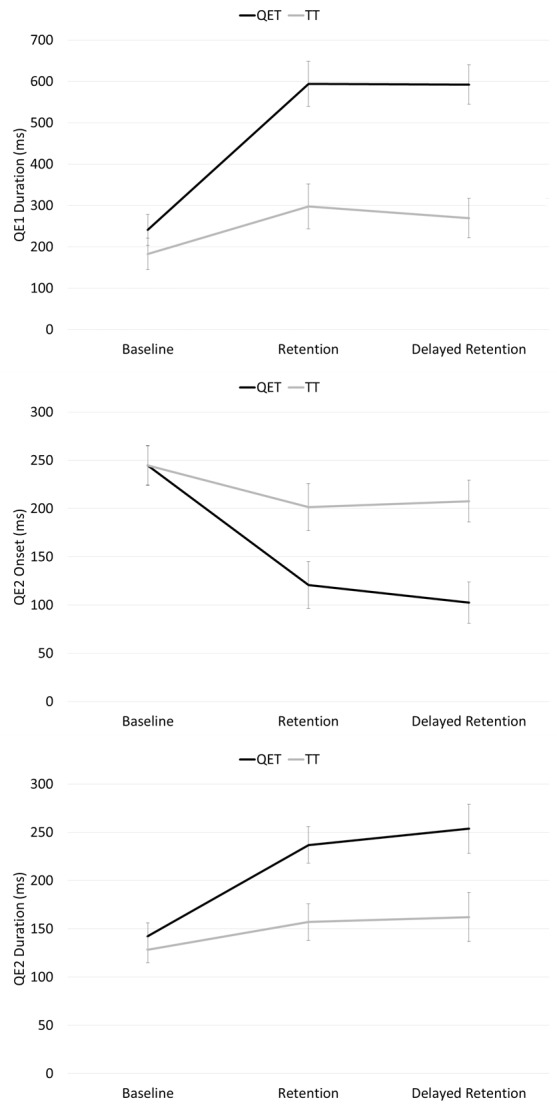
Mean (s.e.m.) QE1 duration (ms; top), QE2 onset (ms; middle) and QE2 duration (ms; bottom) data for Quiet Eye Training (QET) and Technical Training (TT) groups across baseline (QET *n* = 11, TT *n* = 10), retention (QET *n* = 11, TT *n* = 10), and delayed retention tests (QET *n* = 9, TT *n* = 9).

A significant interaction, *F*(2,32) = 5.06, *p* = .012, η_p_^*2*^ = .24, was found for QE1 duration. Post hoc *t*-tests, with an adjusted alpha value for multiple comparisons (0.05/3 = .0167), revealed no significant difference (*p* = .486) between QET (*M* = 241.09, *SD* = 134.48ms) and TT (*M* = 182.99, *SD* = 87.21ms) groups at baseline. However, the QET group had significantly longer (*p* < .001) QE1 aiming durations (*M* = 594.15, *SD* = 150.09ms) at retention compared to the TT group (*M* = 297.80, *SD* = 176.67ms). The significant difference between groups was maintained at delayed retention test where the QET group exhibited significantly longer (*p* < .001) QE1 aiming durations (*M* = 592.56, *SD* = 136.75ms) compared to the TT group (*M* = 269.61, *SD* = 149.21ms; see Fig 2 top).

A significant interaction was also found for QE2 onset, *F*(2,32) = 4.97, *p* = .013, η_p_^*2*^ = .24. Post hoc t-tests, with an adjusted alpha value for multiple comparisons (0.05/3 = .0167), revealed no significant difference (*p* = .878) between QET (*M* = 244.57, *SD* = 71.26ms) and TT groups (*M* = 244.84, *SD* = 48.81ms) at baseline. However, the QET group had significantly earlier (*p* = .005) QE2 onsets (*M* = 120.81, *SD* = 70.19ms) at retention compared to the TT group (*M* = 201.54, *SD* = 75.90ms). The significant difference between groups was maintained at delayed retention test where the QET group exhibited significantly earlier (*p* = .003) QE2 onsets (*M* = 102.36, *SD* = 44.59ms) compared to the TT group (*M* = 207.75, *SD* = 79.23ms; see Fig 2 middle).

A significant interaction was also found for QE2 duration, *F*(2,32) = 3.44, *p* = .045, η_p_^*2*^ = .18. Post hoc t-tests, with an adjusted alpha value for multiple comparisons (0.05/3 = .0167), revealed no significant difference (*p* = .484) between QET (*M* = 142.49, *SD* = 47.29ms) and TT (*M* = 128.39, *SD* = 32.71ms) groups at baseline. However, the QET group had significantly longer (*p* = .001) QE2 durations at retention (*M* = 236.85, *SD* = 43.30ms). compared to the TT group (*M* = 156.96, *SD* = 67.65ms). The difference between QET (*M* = 253.73, *SD* = 55.18ms) and TT (*M* = 162.14, *SD* = 92.67ms) groups at delayed retention tests failed to reach adjusted levels of significance (*p* = .022, see Fig 2 bottom).

### Catching performance

**Fig 3:**
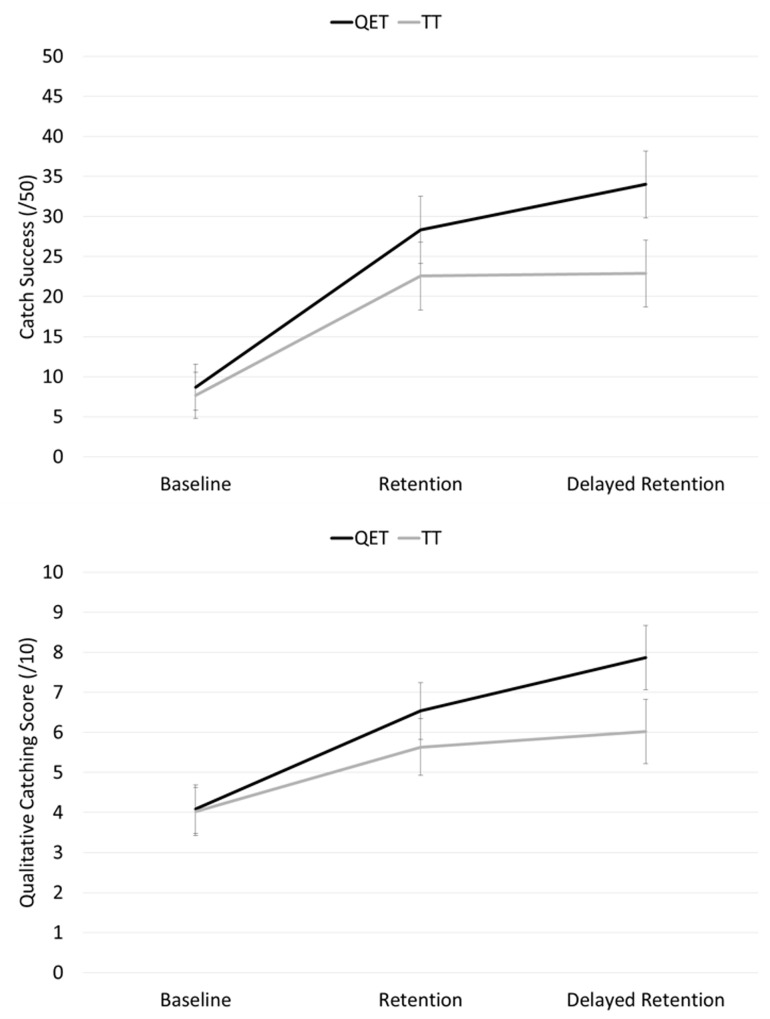
Mean (s.e.m.) number of catches (0–50; top) and catching performance score (0–10; bottom) for Quiet Eye Training (QET) and Technical Training (TT) groups across baseline (QET *n* = 11, TT *n* = 10), retention (QET *n* = 11, TT *n* = 10), and delayed retention tests (QET *n* = 9, TT *n* = 9).

For catch success, a significant main effect was found for test, *F*(2,32) = 44.63, *p* < .001, η_p_^*2*^ = .74, indicating that both groups caught significantly more balls from baseline (*M* = 8.17, *SD* = 8.42) to retention (*M* = 25.44, *SD* = 12.63) test (*p* < .001) and maintained this improvement (*M* = 28.44, *SD* = 913.45) at delayed retention (*p* = .351). The interaction effect was not significant, *F*(2,32) = 2.38, *p* = .108, η_p_^*2*^ = .13, (Fig 3 top).

As the qualitative catching scores were not normally distributed, these data were subjected to natural log transformations prior to analysis. From this, a significant interaction, *F*(2,32) = 3.89, *p* = .031, η_p_^*2*^ = .20, was found. A one way ANOVA revealed no significant between-group differences at baseline (*p* = .413; QET: *M* = 4.10, *SD* = 1.58 vs. TT: *M* = 3.73, *SD* = 2.02) or retention (*p* = .236; QET: *M* = 6.54, *SD* = 2.06 vs. TT: *M* = 5.45, *SD* = 2.30) but the QET group had significantly higher (*p* = .032) qualitative catching scores at delayed retention (QET: *M* = 8.07, *SD* = 1.61 vs. TT: *M* = 5.53, *SD* = 3.01). Within-group post hoc *t*-tests, with an adjusted alpha value for multiple comparisons (0.05/4 = .0125), revealed that the TT group did not significantly improve from baseline to retention (*p* = .028) or from retention to delayed retention (*p* = .529). The QET group significantly improved from baseline to retention (*p* < .001) and maintained this improvement (*p* = .014) at delayed retention (Fig 3 bottom).

### Mediation analysis

Results from mediation with bootstrapping (based on 10,000 sampling rate) indicated that there was a significant indirect effect for QE2 tracking onset (95% confidence interval = 0.01–0.06) in mediating the effect between QE1 aiming duration and catch success.

### Parental questionnaire

#### Likert questions (Part I)

Significant group-based differences were found in the degree to which parents felt their child’s motor coordination had improved, supporting the objective performance data (see Table 4).

**Table 4:** Parental responses given to (Part I) Likert scale questions (means and SD) and (Part II) the open-ended questions (% frequency of parents who made statements) between Quiet Eye Training (QET) and Technical Training (TT) groups.

	QET	TT	CI
***Part I****–Likert Questions*			
Has your child’s throwing and catching improved?	4.38 (.52)	2.83 (.75)	0.91–2.20**
Has your child’s overall coordination improved?	3.88 (.83)	2.67 (1.03)	0.28–2.15*
Has your child’s confidence improved?	4.13 (.64)	3.83 (.75)	-0.40–0.99
Has your child’s attitude towards physical activity improved?	3.88 (.99)	3.00 (1.67)	-0.49–2.25
Has your child chosen to take part in physical activity more often since taking part in this study?	3.88 (.99)	4.00 (1.47)	-1.37–1.13
***Part II****–Themes from Open-ended Questions*			
Motor skill improvements	78%	56%	-0.07–0.17
Improved confidence	89%	67%	-0.07–0.15
Higher predilection for sport	78%	78%	-0.10–0.10
Enjoyment	78%	89%	-0.08–0.12
Social benefits	67%	67%	-0.12–0.12

**p < .01

*p < .05

#### Qualitative comments (Part II)

The result of the content analysis was that we were able to group parents’ comments into five common themes (see Table 4). Frequently the themes of improved confidence, enjoyment, social benefits and predilection for continued participation in sport were reported together (as outlined below):

Confidence / predilection / motor improvement. *“She has definitely improved her confidence and will try more activities*. *Her throwing and catching skill have massively improved and we can see this when we watch her play sport*.*”(Parent of child 10 in QET group)*

Confidence / social benefits. *“Increased confidence from knowing and seeing that he is not the only one who struggles–this has helped his confidence to keep persevering and practicing things that he finds difficult*.*” (Parent of child 5 in QET group)*

Confidence / social benefits. *“Working with other children of the same ability was a unique experience and really beneficial to xxxxx’s confidence*.*” (Parent of child 6 in TT group)*

Enjoyment / social benefits. *“Fun activities really helped to motivated her she also enjoyed talking to similar ability children*.*” (Parent of child 11 in QET group)*

Enjoyment / social benefits / predilection. *“xxxxx has enjoyed meeting children of a similar ability and enjoyed the group session activities……*.*has taken more of an interest in different activities such as cricket and rounders*.*” (Parent of child 7 in QET group)*

Enjoyment / social benefits / confidence. *“Enjoyed it all and really benefited from interacting with similar children*. *Increased her confidence” (Parent of child in 8 QET group)*

Another interesting theme was that parents favourably compared the environment from the study with what their children typically experienced.

“There needs to be more things like this for these children. In normal PE/after school club activities these children feel like they aren’t good enough to play but here they are free to play and feel able to try different activities.” (Parent of child 8 in QET group)

“This felt like more of a coaching club or after school club which I think really helped the children to relax and take part. Normal therapy sessions are totally the opposite of this (i.e boring!).” (Parent of child 12 in QET group)

“He really dislikes his current physical therapy sessions and finds them boring, but these sessions are fun and engaging.” (Parent of child 9 in TT group)

## Discussion

The aim of this study was to explore the efficacy of a group-based QET intervention for improving the catching coordination and success of children with DCD. By combining QET with group-based therapy, we hoped to integrate an intervention that improves skill acquisition in children with poor motor coordination, within an approach that may help ameliorate the psychosocial influence of these motor skill deficits. Our long-term aim was to work towards a holistic intervention for children with DCD that health professionals can use in their therapeutic practice.

In line with our hypotheses and prior research [10,11], QET children learned to aim effectively which enabled them to predict the ball’s bounce point on the wall and track its trajectory for longer (Fig 2). Mediation analysis showed that longer aiming durations (QE1) predicted an earlier onset of tracking the ball prior to catching (QE2) which predicted catching success [9]. In short, this optimization of gaze provided children with earlier information on which to prepare the interceptive catch attempt [8, 9]. Interestingly, after completing the QET intervention the QE durations used by these DCD children when aiming (QE1 mean 592ms) and tracking the ball (QE2; mean 254ms) were almost identical to the durations that highly skilled children used to aim (QE1 mean 496ms) and track the ball (QE2 mean 255ms) in the same task [8].

These changes in visuomotor control were also reflected in children’s catching performance. While the improvement between groups in the dichotomous measure of catch success was not significantly different (despite the QET group improving by 51% compared to the TT improvement of 31%) the more sensitive catching score measure did show significant differences as we hypothesised. Specifically, QET individuals significantly improved their catching technique from baseline to retention and maintained this improvement at the delayed retention test. Conversely, the TT individuals did not significantly improve their catching technique throughout the experiment (see Fig 3). In effect, this meant that after training, the QET children were able to catch the ball cleanly with both hands before securing it against their body (mean score of 8), whereas the TT group were catching the ball by trapping it against their body with their arms, rather than grasping it with their hands (mean score of 6). Future analyses will attempt to quantify these changes in terms of patterns of motor coordination and muscular activity.

Results from both the gaze and performance data highlight some important implications. First, regardless of the training intervention, all children were able to improve on this interceptive motor task; highlighting the important distinction that DCD should be considered as impaired motor proficiency rather than a fundamental inability to learn motor skills [23]. Second, DCD children can learn to make more functional eye movements that can directly compensate for the oculomotor atypicalities associated with this disorder [6]. Third, QET instructions appear to deliver more effective retention of interceptive skill than typical, explicit instructions focusing on movement control. These benefits are likely due to reduced demands on cognitive load supporting motor control [24] and the promotion of a more implicit motor learning environment [25]: Rather than having to consciously think about the complex control of multiple limbs, these are guided implicitly once gaze control is optimised. Previous research has identified that implicit motor learning is an effective strategy for children with DCD [26], perhaps due to how it overcomes deficits in working memory resources [27]. Overall, while the specific mechanisms underpinning the QET benefits warrant further investigation [28], there appears to be clinical utility in exploring the application of QET to other tasks where children with DCD experience difficulties.

Interestingly, the improvements in motor coordination displayed by the children were reflected in the perceptions of their parents. Parents of children in the QET group reported that their children had improved their throwing and catching ability and overall coordination to a greater extent than parents of TT children (see Table 4). We also found that parents of children in both groups reported that the intervention had improved their child’s confidence, social behaviours and predilection for sport as we hypothesised. Despite these promising findings, we must add a cautionary caveat here highlighting that we used an unvalidated parental feedback questionnaire and a relatively small sample size. Future research should use validated inventories (e.g., the Children Performance Skills Questionnaire [29]) with a larger sample of children in order to explore these findings further. Additionally, parental perceptions of their child’s predilection for sport or physical activity could also be more objectively measured (e.g., accelerometers) in future work in order to build on these initial results. Despite these limitations, there does appear to be considerable psychosocial benefits of group-based training irrespective of the specific content of the session and the extent of any motor improvements [14].

The positive results of the study lead us to suggest a number of cautionary applied recommendations to enhance the therapeutic experience of children with DCD. First, we wish to echo previous recommendations that interventions for children with DCD must aim to improve both the motor deficits and psychosocial consequences of the disorder [17]. Applied practitioners may benefit from developing innovative ways to incorporate group-based therapy sessions. While the clinical setting may not be the best place to achieve this aim, other organisations such as local councils, schools, national governing sporting bodies and professional sports clubs may have a part to play in supporting such initiatives. Either way, a group-therapy approach is something that children with DCD and their parents find beneficial and there is currently a lack of provision for these types of activities, particularly in the UK.

Second, it seems that instructions that optimise the visual attention of children with DCD have real and durable benefits for motor skill attainment in this catching task. As children with DCD generally struggle to develop effective learning strategies on their own–they generally persist with ineffective ones [23]–equipping them with a strategy that has been proven to expedite the learning process in a number of tasks and populations seems a logical step for applied practitioners to take. Understandably, to achieve this will take a greater collaboration between the scientists who are familiar with these methods and the practitioners who will be at the forefront of their delivery.

In conclusion, we aimed to develop a holistic intervention for children with DCD that would not only alleviate some of the visuomotor deficits that they experience but also improve some of the psychosocial consequences of the disorder. By combining an intervention (i.e., QET) that has been shown to improve motor learning in children with DCD within a group-based therapy framework, we believe that this aim has been achieved. Future research in now needed to establish the feasibility and practicality of incorporating these ideas into applied therapeutic practice.

## Supporting information

S1 Fig The CONSORT checklist. (DOC)

S1 File The original study protocol taken from NHS ethics application. (DOCX)

S2 File Instructional videos. Summarising the instructions given to the QET and TT groups.
(ZIP)
