# Supplementary material for: A randomized controlled trial of a group-based gaze training intervention for children with Developmental Coordination Disorder
Source: PLoS One. 2017 Feb 10;12(2):e0171782. doi: 10.1371/journal.pone.0171782 (PMC5302797; doi:10.1371/journal.pone.0171782)
Supplement: S1 File — (DOCX) [file pone.0171782.s002.docx]

**Study Protocol taken from NHS ethics application 15/NW/0279**

Each participant will be invited to attend four phases of this study. The first phase is the Assessment where the participant (with a parent or guardian) will visit the laboratory at Liverpool Hope University. Here they will be given detailed information about the study and protocol and asked to sign the ethics consent forms. Parents/guardians will be asked to complete an information form about the child's condition(s) and an ADHD checklist.

The child will then complete the 5 of the tasks of the Movement Assessment Battery (2nd Edition) as specified by the battery (Henderson et al., 2007). The child will then be fitted with an ASL gaze registration system, EMG electrodes on the forearm and shoulder, kinematic markers will be placed on their arm, and shoulder and the equipment will be calibrated. The child will then complete the remaining catching and balance tasks (task 4, 7 & 8) complete additional trials (40) of the throwing and catching task to ascertain their baseline performance level. Two further short catching tasks will also be completed at this stage. This assessment phase will take between 40-60mins to complete. The researchers will then pseudo-randomly allocate the child to either a gaze training (QET) or movement training (TT) intervention group.

The second phase of the study is the training phase and will take place shortly after the assessment phase. This is a 6-week training protocol where the participants will attend weekly training sessions held at Liverpool Hope University Sports Hall (depending on recruitment these courses may occur at different times or more than one course may be required). The training will include a combination of videos and exercises targeted at explicitly teaching the children the required technique. Each training session will take approximately 60mins and will consist of up to 15 children. At least two qualified coaches/researchers will be present throughout all sessions.

One week after the training phase is completed the participant will return to the laboratory at Liverpool Hope University for the first retention phase (R1). Here the children will attend individually and will complete all the measures taken during the assessment phase.

Finally, 6 weeks after the first retention phase, when a period of no practice has taken place, the children will come into the laboratory for a final delayed retention phase (R2). Again, this will repeat the assessment phase measures. On the conclusion of this phase, the children and their parents will be thanked and fully debriefed about the study.

The data analysis procedures will include a series of ANOVAs to explore between-group differences.

****This is the full and original protocol (section A13) from the IRAS application form.**
